# Supplementary material for: Demographic Characteristics and Economic Burden of Clostridioides difficile Infection in Korea: A Nationwide Population-Based Study after Propensity Score Matching
Source: Antibiotics (Basel). 2024 Jun 10;13(6):542. doi: 10.3390/antibiotics13060542 (PMC11201190; doi:10.3390/antibiotics13060542)
Supplement: Supplementary file 1 [file antibiotics-13-00542-s001.zip › antibiotics-3025335-supplementary.pdf]

antibiotics

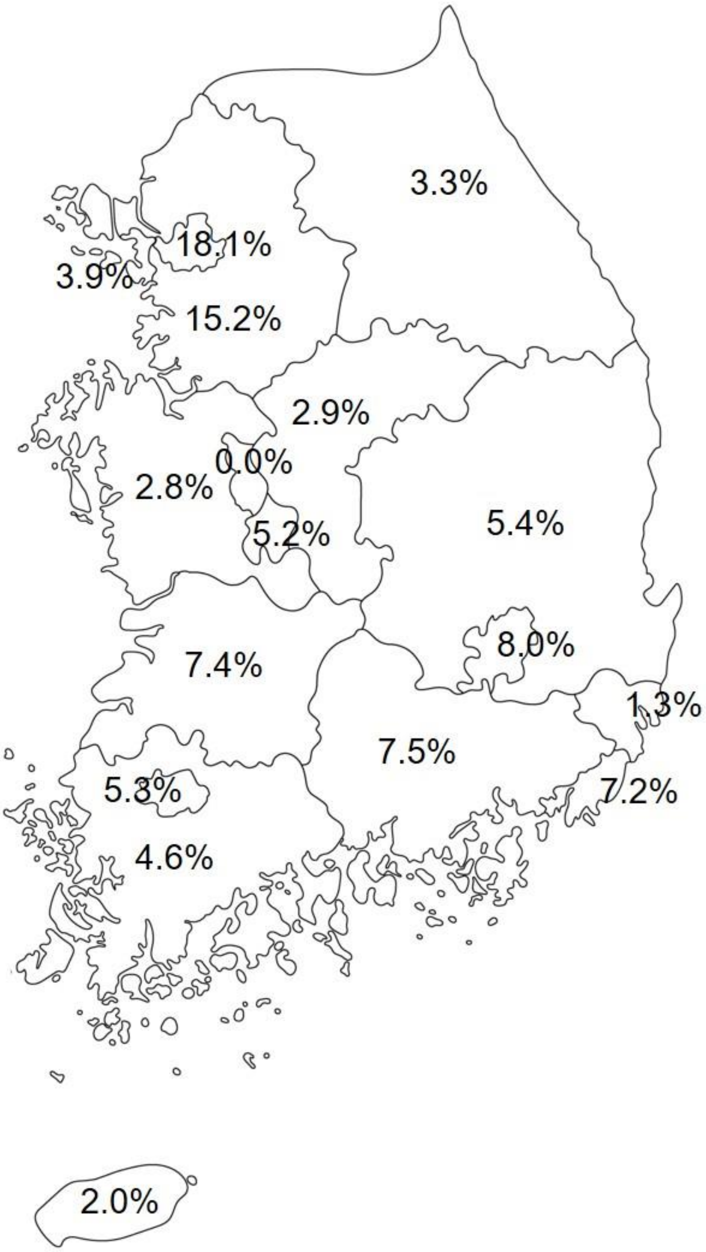

Figure S1. Nationwide regional distribution of CDI occurrence.

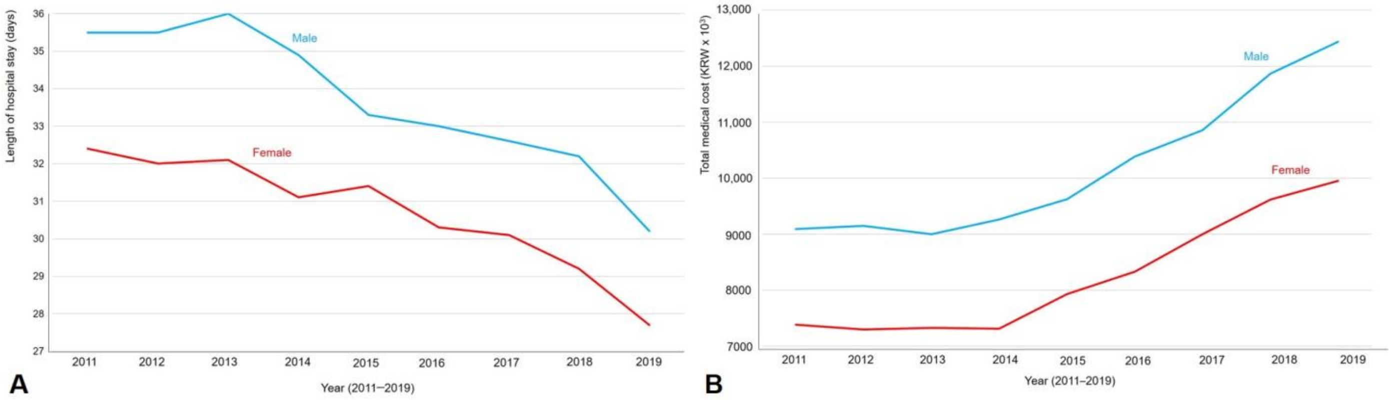

Figure S2. Length of hospital stay (**A**) and total medical costs (**B**) according to sex. The length of hospital stay is significantly decreased, while the total medical cost is significantly increased in both sexes (both $P < 0.001$).

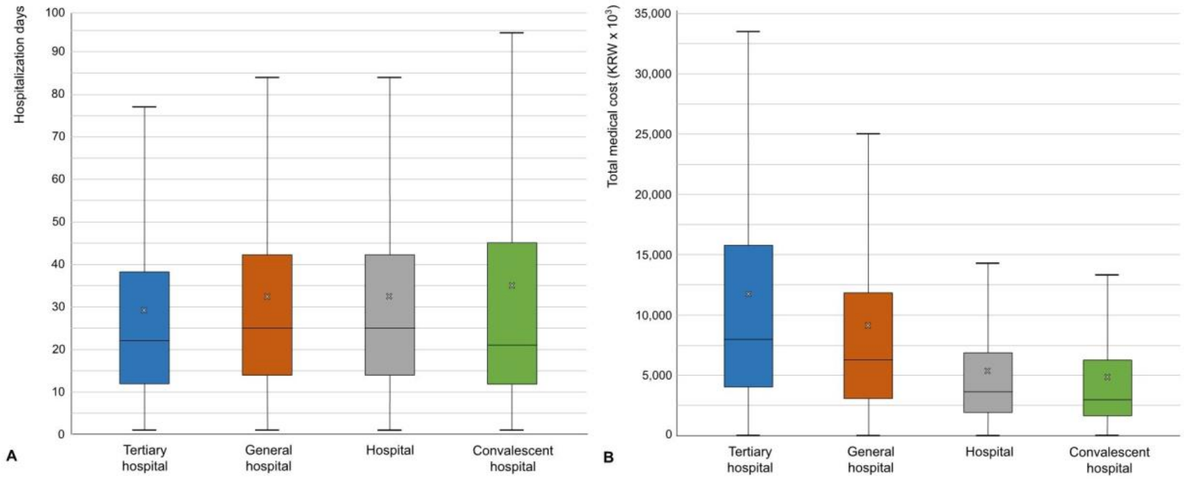

Figure S3. Length of hospital stay (**A**) and total medical costs (**B**) according to type of hospitals. Length of hospital stay was significantly lower in tertiary hospitals, but the total medical costs were significantly higher in tertiary hospitals than in other types of hospitals (both $P < 0.001$).

Table S1. Duration of hospital stay and total medical costs associated with CDI using the regression model.

		Model 1	Model 2	Model 3
Length of hospital stay (days)				
Independent variable	CDI	21.9	15.1	15.3
	P value	< 0.001	< 0.001	< 0.001
R^2		0.2591	0.4367	0.4214
Adjusted R^2		0.2591	0.4367	0.4214
P in regression model		< 0.001	< 0.001	< 0.001
Total medical cost (×10^3 won)				
Independent variable	CDI	7603.8	3436.5	3413.1
	P value	< 0.001	< 0.001	< 0.001
R^2		0.2765	0.6352	0.6337
Adjusted R^2		0.2765	0.6351	0.6337
P in regression model		< 0.001	< 0.001	< 0.001

Model 1 does not use control variables; Model 2 uses all variables (sex, age group, year of hospitalization, type and region of hospital, comorbidity index, and maximum medical cost) as control variables, without considering matching results; and Model 3 assumes sex, age group, and year of hospitalization are the same as matching.
